# Oxytocin Signaling Bidirectionally Modulates Serotonergic and Dopaminergic Neurons in the Dorsal Raphe Nucleus

**DOI:** 10.1523/ENEURO.0182-26.2026

**Published:** 2026-11-02

**Authors:** Ja Eun Choi, Yoon Kyoung Kim, Yongmin Sung, Dong Il Choi, Jooyoung Kim, Chaery Lee, Jisu Lee, Nam-Kyung Yu, Han Kyoung Choe, Bong-Kiun Kaang

**Affiliations:** ^1^School of Biological Sciences, Seoul National University, Seoul 08826, South Korea; ^2^Department of Brain Sciences, Daegu Gyeongbuk Institute of Science and Technology (DGIST), Daegu 42988, South Korea; ^3^Center for Memory and Glioscience, Institute for Basic Science (IBS), Daejeon 34126, South Korea

**Keywords:** dopamine, dorsal raphe nucleus, oxytocin, paraventricular nucleus, serotonin, social behavior

## Abstract

The involvement of oxytocin in social behavior is well established, yet its interaction with neuromodulatory systems, such as serotonin and dopamine, remains incompletely understood. Here, we utilized immunohistochemistry, anatomical tracing, chemogenetic, and ex vivo electrophysiological approaches primarily in male mice, with female mice used only for an additional anatomical tracing control experiment, investigating how oxytocin modulates serotonergic and dopaminergic neuronal populations. Employing the dual-eGRASP tool, we visualized oxytocinergic projections from the paraventricular nucleus (PVN) to the serotonergic and dopaminergic neurons in dorsal raphe nucleus (DRN). Blocking oxytocin receptors in the DRN attenuated social interaction behavior. Additionally, analysis of c-Fos levels and neuronal excitability revealed that oxytocin inhibits serotonergic neurons while activating dopaminergic neurons in the DRN. Our findings suggest a bidirectional regulatory role of oxytocin signaling in the DRN serotonergic and dopaminergic neurons.

## Significance Statement

Social behavior relies on interactions among neuromodulatory systems, yet how oxytocin communicates with serotonin and dopamine circuits remains unclear. We identify the dorsal raphe nucleus (DRN) as a functionally relevant target of hypothalamic oxytocin neurons during social approach. Using dual-eGRASP, we visualize contacts from paraventricular nucleus oxytocin neurons onto both serotonergic and dopaminergic DRN. Blocking oxytocin receptors in the DRN reduces social interaction without affecting locomotion. Moreover, oxytocin suppresses serotonergic neuronal activity while increasing dopaminergic neuronal activation, revealing opposing control of two prosocial neuromodulatory populations within the same region. These findings provide a circuit framework for how oxytocin coordinates social behavior through bidirectional modulation of dorsal raphe neuromodulators.

## Introduction

The complex social behaviors are driven by a finely tuned interplay of fast, focused neurotransmission and slow, widespread neuromodulation within specialized neural circuits (Wei et al., 2021; Wu and Hong, 2022; Li et al., 2025). Among the diverse array of intercellular neuromodulators, oxytocin, dopamine, and serotonin are often considered as master regulators that process distinct dimensions of social cognition, motivation, and emotional states (Dölen et al., 2013; Gunaydin et al., 2014; Walsh et al., 2018; Matthews and Tye, 2019; Froemke and Young, 2021; Grinevich and Neumann, 2021; Jang et al., 2023). This neuromodulatory triad is particularly critical for driving social approach—the fundamental behavioral drive to initiate proximity and investigate a conspecific. Social approach consistently increased the activities of oxytocin, dopamine, and serotonin neurons, whereas functional impairment of any of these neurons via optogenetic, chemogenetic, or genetic disturbance inhibited the social approach behaviors (Gunaydin et al., 2014; Resendez et al., 2020; Jang et al., 2023). Notably, experimental activation of oxytocin neurons or dopamine neurons alone suffice to increase social approach (Gunaydin et al., 2014; Resendez et al., 2020). These suggest that the function of these key neuromodulators in social approach cannot simply be considered as three independent system but rather be regarded as cooperative interacting system. However, despite their clear cooperative roles in governing social approach, the precise mechanisms through which these three systems converge and cross-regulate one another within specific brain centers remain unclear.

Oxytocin neurons in the paraventricular nucleus (PVN^OXT^) send brain-wide projection to modulate various downstream neural circuits during social behavior. Based on the extensive mapping of PVN^OXT^, one downstream region is the dorsal raphe nucleus (DRN; Weissbourd et al., 2014; Son et al., 2022). The DRN contains heterogeneous neuronal populations, including serotonergic, dopaminergic, and GABAergic neurons, that contribute to distinct aspects of social and affective behavior (Huang et al., 2019). Both dopaminergic and serotonergic neurons in the dorsal raphe (DRN^DA^ and DRN^SERT^, respectively) are critical in triggering social behaviors and mediating social context (Dölen et al., 2013; Matthews et al., 2016; Choi et al., 2022). Previous studies suggest a functional interaction between oxytocin and raphe serotonergic signaling, although the direction of this regulation remains incompletely understood. Oxytocin has been reported to facilitate serotonin release in the median raphe nucleus (Yoshida et al., 2009) and to excite DRN serotonergic neurons through oxytocin receptors in ex vivo recordings (Oubraim et al., 2023). In contrast, oxytocin-induced changes in serotonin receptor binding in the human DRN and limbic regions have been interpreted as consistent with reduced serotonin signaling (Mottolese et al., 2014). Moreover, oxytocin receptor expression has been detected in TH-expressing neurons in the DRN, suggesting that oxytocin may also influence DRN dopaminergic neurons (Grieb and Lonstein, 2021). However, whether PVN^OXT^ neurons anatomically engage both DRN^SERT^ and DRN^DA^ populations, and how oxytocin signaling regulates these two neuromodulatory systems during social behavior, remains unclear. Here, we investigated the hypothesis that oxytocin signaling in the DRN coordinates serotonergic and dopaminergic neuronal populations to regulate social approach behavior.

In this study, we examined how PVN^OXT^ signaling engages serotonergic and dopaminergic populations in the DRN to regulate social approach behavior. Using the dual-eGRASP tool (Choi et al., 2018, 2021, 2022; Lee et al., 2023; Kim et al., 2024), a fluorescence reconstitution-based tool that visualizes contact sites between genetically defined presynaptic and postsynaptic neuronal populations, we visualized the contact sites between PVN^OXT^ and DRN^DA^ as well as DRN^SERT^. Blocking oxytocin receptors within the DRN via antagonist infusion resulted in attenuation of social interaction levels. Additionally, we observed opposite effects on neuronal activity within the DRN^SERT^ and DRN^DA^ populations following oxytocin administration, with a decrease in activity in DRN^SERT^ neurons and an increase in activity in DRN^DA^ neurons. These effects may involve local DRN circuit mechanisms. Together, these findings identify the DRN as a functional downstream target of PVN^OXT^ neurons and reveal bidirectional regulation of dorsal raphe serotonergic and dopaminergic systems by oxytocin signaling.

## Materials and Methods

### Animals

OXT::Cre (JAX 024234), SERT::Cre (JAX 014554), TH::Cre (JAX 008601), and VGAT::Cre (JAX 028862) were maintained in a C57BL/6J background. *Ai14* reporter mice (JAX 007914) were used to visualize neurons in a Cre-dependent manner. Unless otherwise specified in the corresponding figure legends, all experiments were performed using group-housed male mice. Mice were maintained under a 12 h light/dark cycle, with lights on at 09:00, defined as zeitgeber time 0 (ZT0), and had access to food and water *ad libitum*. All animal procedures were performed in accordance with the regulations of the Seoul National University Institutional Animal Care and Use Committee.

### Stereotaxic surgery

Mice were anesthetized with a ketamine/xylazine solution and positioned on a stereotaxic apparatus (Stoelting). The virus mixture was injected into target regions through a 32-gauge needle with a Hamilton syringe at a rate of 0.1 µl/min, and the total injection volume per site was 0.3–0.5 µl. The needle tip was positioned 0.05 mm below the target coordinate right before the injection for 2 min. After the injection was completed, the needle remained in place for an additional 7 min and was withdrawn slowly. The stereotaxic coordinates for each target site were as follows: PVN (AP: −0.3/ ML: ±0.2/ DV: −5.2 mm) and DRN (AP: −4.2/ ML: 0/ DV: −3.2 mm).

### Dual-eGRASP

Dual-eGRASP was used to visualize putative contact sites between PVN^OXT^ neurons and DRN^SERT^ or DRN^DA^ neurons. Viral constructs used for dual-eGRASP labeling were previously validated and described in detail (Choi et al., 2018, 2021, 2022; Lee et al., 2023). For visualization of PVN^OXT^ axons in the DRN, the pre-eGRASP construct was modified to include mScarlet fluorescence, allowing simultaneous identification of presynaptic axons and GRASP puncta. Perfused brains were fixed with 4% paraformaldehyde in PBS overnight at 4°C and dehydrated in 30% sucrose in PBS for 2 d at 4°C. Brain slices (50 µm) were mounted in Vectashield mounting medium (H-1000; Vector Laboratories). Leica SP8 confocal microscope with 63× objective was used for imaging (Leica Microsystems). Processing the confocal image were performed using IMARIS (Bitplane) software.

### Immunohistochemistry

Brains were postfixed in 4% PFA at 4°C overnight and transferred to 30% sucrose for 2 d at 4°C. Brains were embedded in optimal cutting temperature mounting medium, and 40 µm sections were cut using a cryostat (Leica Microsystems). Primary antibody rabbit anti-c-Fos (226003; Synaptic Systems) were diluted to 1:1,000 in blocking solution and incubated for 24 h at 4°C. Secondary antibodies in the blocking solution were incubated for 2 h at room temperature, and sections were mounted with Vectashield mounting medium. Images were viewed under a Leica SP8 confocal microscope.

### Electrophysiology

Adult mice were anesthetized with isoflurane, and their brains were quickly dissected. *N*-methyl-d-glucamine (NMDG) solution (in mM: 93 NMDG, 2.5 KCl, 1.2 NaH_2_PO_4_, 30, NaHCO_3_, 20 HEPES, 25 glucose, 5 sodium ascorbate, 2 thiourea, 3 sodium pyruvate, 10 MgSO_4_, 0.5 CaCl_2_) was used during brain slicing and recovery. Transverse slices (250 µm) were prepared in an ice-cold NMDG solution with a vibratome and then recovered in a 32°C NMDG solution for 6–7 min. After at least 1 h of recovery in ACSF (in mM: 124 NaCl, 2.5 KCl, 1 NaH_2_PO_4_, 25 NaHCO_3_, 10 glucose, 2 CaCl_2_, and 2 MgSO_4_), the slice was placed in the recording chamber perfused with 32°C ACSF. To obtain reliable recordings, patched cells were allowed to stabilize for at least 3 min, and resting membrane potential was then recorded with no injected current. Only cells with a change in access resistance <20% were included in the analysis. The recording pipettes (2–4 MΩ) were filled with an internal solution containing the following (in mM): 145 K-gluconate, 5 NaCl, 10 HEPES, 1 MgCl_2_, 0.2 EGTA, 2 MgATP, and 0.1 Na_3_GTP (280–300 mOsm, adjusted to pH 7.2 with KOH). Oxytocin (1 µM) was applied via bath perfusion for 7–10 min. The neuronal excitability before oxytocin application was compared with its effect postapplication.

### Chemogenetic manipulations

The designer drug clozapine-*N*-oxide (CNO, 10 mg/kg for inhibition, i.p., Hello Bio) was administered 40 min before the behavioral tasks.

### Intracranial drug delivery

Twenty-six-gauge guide cannulas were implanted into the DRN (AP: −4.25/ ML: +1.25/ DV: −3.05) at a 20° angle. After recovery, the mice were infused with 0.5 µl of L-368,899 10 mM (Tocris Bioscience) or saline vehicle. The 30-gauge needle was injected 0.2 mm lower than the guide cannula. After the volumes were injected, it was left in place for 3 min to allow diffusion. Three-chamber tests started 30 min after saline or drug infusion.

### Three-chamber test

During the habituation session, mice were allowed to freely move in the apparatus for 10 min. After the habituation, an unfamiliar juvenile mouse (stranger) was placed under one cup and an object was placed under the other cup. Then the test mice were recorded for the preference to the sex-matched unfamiliar juvenile mouse for 10 min, which is the test session. The time of sniffing to each cup (stranger and object) was analyzed. The difference score was calculated by subtracting time spent sniffing the stranger by the time of sniffing the object, which was regarded as sociability. EthoVision software generated the heat maps and analyzed the velocity and total distance moved.

## Results

The PVN is a crucial brain region involved in social behavior (Wei et al., 2021; Wu and Hong, 2022; Li et al., 2025). Consistent with previous reports, mice engaged in social interactions exhibited increased activity in the PVN, as indicated by elevated c-Fos levels (Fig. 1*A*; two-tailed Welch's unpaired *t* test, *p* = 0.0224). Among the cell types in the PVN, the oxytocinergic neuronal population (PVN^OXT^) is known to be essential for social behavior (Froemke and Young, 2021; Grinevich and Neumann, 2021). To investigate this further, we utilized OXT::Cre mice and expressed Gi-coupled DREADD (designer receptor exclusively activated by designer drugs) hM4Di in PVN^OXT^ neurons to chemogenetically inhibit them during social behavior (Fig. 1*B*). CNO-treated mice exhibited decreased social interaction compared with the saline-injected group, as measured by the time spent interacting with a novel stranger mouse relative to an empty cup (Fig. 1*C*,*D*; two-way RM ANOVA, saline/CNO effect *p* = 0.0006, object/stranger effect *p* = 0.0278, saline/CNO × object/stranger interaction *p* = 0.0145; post hoc Šídák's multiple-comparisons test: saline, object vs stranger, *p* < 0.001; CNO, object vs stranger, ns; stranger, saline vs CNO, *p* < 0.01). The difference score was significantly smaller following CNO treatment compared with saline treatment (Fig. 1*E*; two-tailed paired *t* test, *p* = 0.0489). This decrease in social interaction was not accompanied by changes in locomotor activity, as assessed by total distance moved and velocity during the test session (Fig. 1*F*; two-tailed paired *t* test, distance moved *p* = 0.3104, velocity *p* = 0.2700). In mCherry-expressing control mice, CNO treatment did not alter social preference (Fig. 1*G*; two-way RM ANOVA, saline/CNO effect *p* = 0.2008, object/stranger effect *p* < 0.0001, saline/CNO × object/stranger interaction *p* = 0.2682; post hoc Šídák's multiple-comparisons test: saline, object vs stranger, *p* < 0.01; CNO, object vs stranger, *p* < 0.001). The difference score (Fig. 1*H*; two-tailed Welch's unpaired *t* test, *p* = 0.2920) and locomotor activity (Fig. 1*I*; two-tailed Welch's unpaired *t* test, distance moved *p* = 0.6699, velocity *p* = 0.6697) did not differ between saline- and CNO-treated mCherry-expressing control mice.

**Figure 1.**
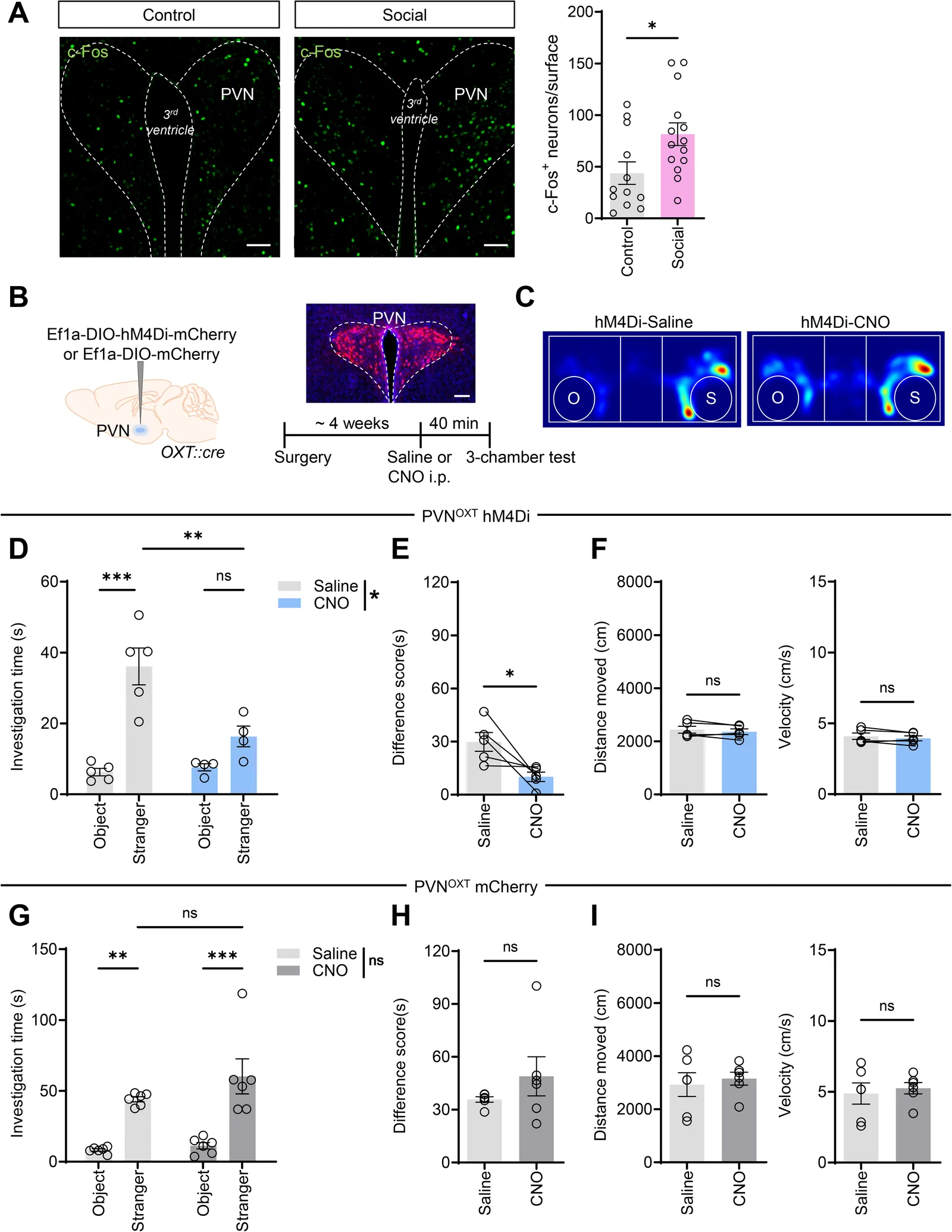
PVN oxytocinergic neurons are required for social approach. ***A***, Representative images of c-Fos expression in the PVN with increased levels observed in the social interacted group compared with controls (social group, *n* = 14 slices from 6 mice; control group, *n* = 12 slices from 4 mice). Scale bars, 100 µm. ***B***, Virus injection site, chemogenetic receptor expression in the PVN, and experimental scheme. Blue, DAPI; red, hM4Di-mCherry. Scale bars, 100 µm. ***C***, Representative heat maps from saline- and CNO-treated hM4Di-expressing mice during the three-chamber test. ***D***, Investigation time toward the object and stranger in saline- and CNO-treated hM4Di-expressing mice. ***E***, Difference scores, calculated as described in the Materials and Methods, for saline- and CNO-treated hM4Di-expressing. ***F***, Distance moved and velocity in saline- and CNO-treated hM4Di-expressing mice. ***G***, Investigation time toward the object and stranger in saline- and CNO-treated mCherry-expressing control mice. ***H***, Difference scores for saline- and CNO-treated mCherry-expressing control mice. ***F***, Distance moved and velocity in saline- and CNO-treated mCherry-expressing control mice.

Then, we asked which brain region is involved in the downstream of PVN^OXT^ activation in social approach behavior. The DRN is one of the brain regions that show robust oxytocin receptor expression (Mitre et al., 2016). We hypothesized that blocking the oxytocin receptor signaling in the DRN would display an attenuation in the social approach behavior. Direct infusion of oxytocin receptor antagonist (OXT-A) into the DRN, decreased the social interaction (Fig. 2*A*–*C*; Fig. 2*B*, two-way RM ANOVA, saline/OXT-A effect *p* = 0.0021, object/stranger effect *p* = 0.0098, saline/OXT-A × object/stranger interaction *p* = 0.0254; post hoc Šídák's multiple-comparisons test: saline, object vs stranger, *p* < 0.01; OXT-A, object vs stranger, ns; stranger, saline vs OXT-A, *p* < 0.01; Fig. 2*C*, two-tailed Welch's unpaired *t* test, *p* = 0.0255). The OXT-A treatment into the DRN did not affect the overall locomotion of the mice (Fig. 2*D*; two-tailed Welch's unpaired *t* test, distance moved *p* = 0.2715, velocity *p* = 0.2763). Thus, these results suggest that oxytocin signaling in the DRN is required for social approach behavior.

**Figure 2.**
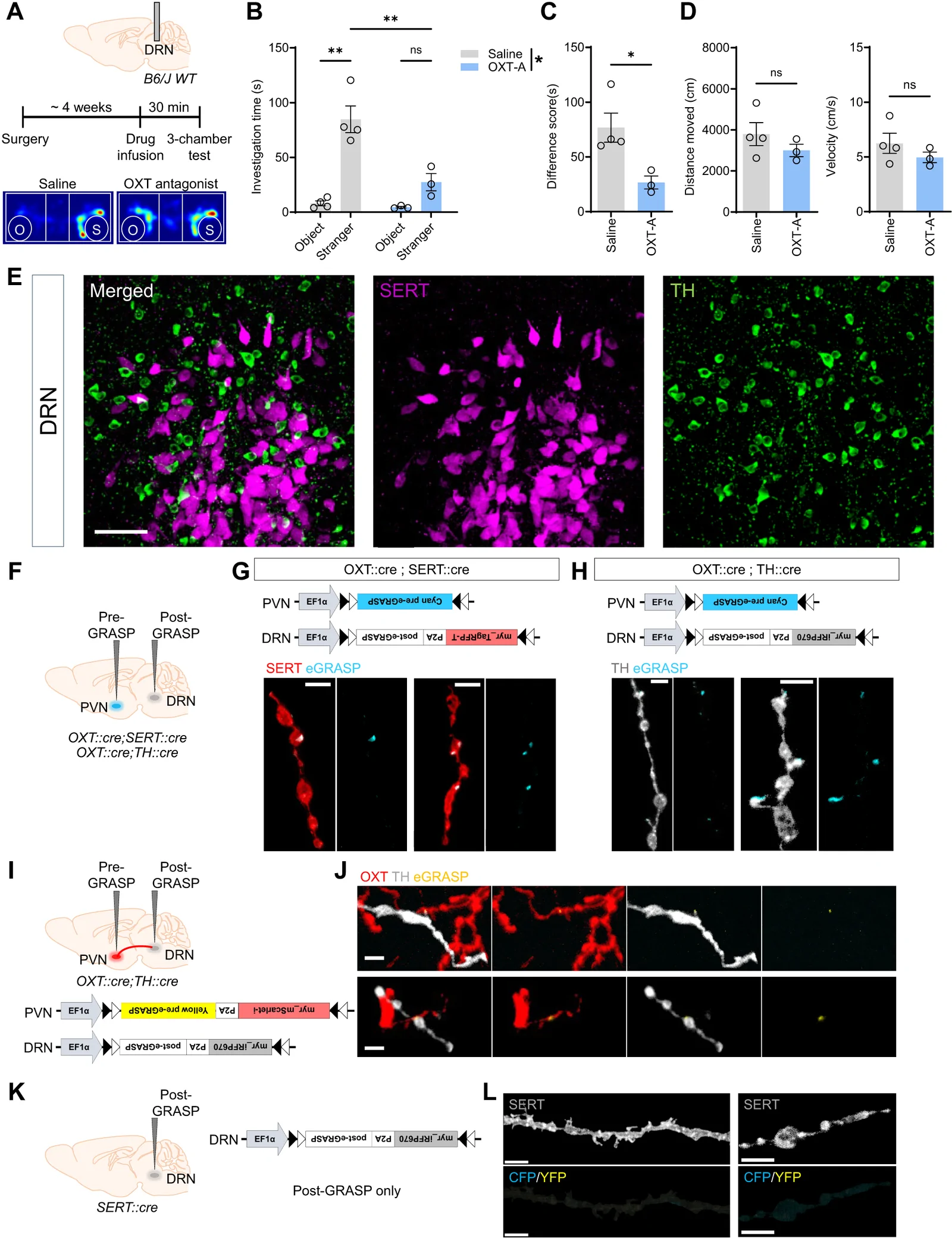
The DRN^SERT^ and DRN^DA^ are downstream of PVN^OXT^. ***A***, Schematic for oxytocin receptor antagonist infusion, experimental timeline, and representative heat maps of saline- or oxytocin antagonist (OXT-A)-infused mice during three-chamber test. ***B***, Investigation time toward the object and stranger in saline and OXT-A-infused mice (saline, *n* = 4 mice; OXT-A, *n* = 3 mice). ***C***, Difference scores in saline- and OXT-A-infused mice. ***D***, Distance moved and velocity during three-chamber test. ***E***, Distinct neuronal population of serotonergic (magenta) and dopaminergic (green) neurons in the DRN. Scale bar, 50 µm. ***F***, Experimental illustration of viral injections for dual-eGRASP labeling. ***G***, Viral constructs for eGRASP and representative images showing oxytocinergic contact sites (cyan, eGRASP) on serotonergic neurons (red, TagRFP). *n* = 6 slices from 3 mice. Scale bars, 5 µm. ***H***, Virus constructs eGRASP and representative images showing oxytocinergic contact sites (cyan, eGRASP) on dopaminergic neurons (gray, iRFP). *n* = 6 slices from 3 mice. Scale bars, 5 µm. ***I***, Experimental illustration of viral injections and the eGRASP constructs. ***J***, Representative images showing PVN^OXT^ axons (red, mScarlet) contacting DRN^TH^ neurons (gray, iRFP) with the contact sites (yellow, eGRASP). *n* = 6 slices from 3 mice. Scale bars, 5 µm. ***K***, Experimental illustration of viral injection and the post-eGRASP construct. ***L***, Representative control images showing the absence of eGRASP signal on serotonergic neurons when the pre-GRASP construct was omitted. Female mice were used; *n* = 5 slices from 2 mice. Scale bars, 5 µm.

To explore the anatomical associations between oxytocinergic neurons and serotonergic or dopaminergic neurons in the DRN, we further investigated their anatomical associations. The DRN contains heterogeneous neuronal populations, including the serotonergic and dopaminergic neurons (Huang et al., 2019; Choi et al., 2022). In line with previous reports, we observed that the serotonergic and dopaminergic neurons were distinctly distributed in the DRN in a nonoverlapping manner (Fig. 2*E*). We asked whether these distinct populations receive anatomical inputs from PVN^OXT^. By employing dual-eGRASP technology, which captures the contacts between presynaptic and postsynaptic neurons (Choi et al., 2018, 2021, 2022; Lee et al., 2023), we examined the anatomical associations of PVN^OXT^ neurons with DRN^SERT^ and DRN^DA^ neurons. OXT::Cre mice were crossed with either SERT::Cre or TH::Cre mice in order to label each cell type in a Cre-dependent manner (Fig. 2*F*). We observed cyan-GRASP puncta at putative contact sites between oxytocinergic neurons and both serotonergic and dopaminergic neurons (Fig. 2*G*,*H*). Given the absence of axon visualizations in previous dual-eGRASP constructs, we made modifications to the pre-GRASP construct to enable axon visualization using mScarlet fluorescence (Fig. 2*I*). By applying this modification to the OXT::Cre; TH::Cre double-Cre line, we visualized PVN^OXT^ axons in the DRN with mScarlet, identified DRN^DA^ with iRFP, and observed the contact sites as yellow-GRASP puncta (Fig. 2*J*). When the post-GRASP construct was injected into the DRN of SERT::Cre mice without the pre-GRASP construct in the PVN, no eGRASP signal was observed on serotonergic neurons (Fig. 2*K*,*L*). Together, these findings provide anatomical evidence supporting putative contact sites between PVN^OXT^ axons and both DRN^SERT^ and DRN^DA^ neurons. This anatomical arrangement suggests that oxytocinergic inputs from the PVN are positioned to influence both serotonergic and dopaminergic neuromodulatory populations within the DRN.

Our next objective was to understand how oxytocin influences the neuronal activity across distinct DRN populations. We first analyzed the number of c-Fos-positive neurons in different neuronal populations within the DRN in response to the exogenous oxytocin administration. The administered oxytocin dosage was determined based on previous studies demonstrating its effects (Tan et al., 2019). In the serotonergic population of the DRN, oxytocin decreased neuronal activity, as we observed a reduction in c-Fos-positive neurons compared with saline-injected mice (Fig. 3*A*; two-tailed, Mann–Whitney test, *p* = 0.0101). Conversely, oxytocin increased neuronal activity in both dopaminergic and GABAergic populations of the DRN (Fig. 3*B*,*C*; two-tailed, Mann–Whitney test, dopaminergic *p* = 0.0344, GABAergic *p* = 0.0003). These opposing changes across serotonergic, dopaminergic, and GABAergic populations suggest that oxytocin may engage distinct local circuit mechanisms within the DRN. To further explore oxytocin's effects, we conducted ex vivo patch-clamp recordings on serotonergic and dopaminergic neurons, comparing their responses before and after oxytocin perfusion. Oxytocin application showed a trend toward reducing the number of generated spikes across current injections and significantly increased the rheobase in DRN^SERT^ neurons, while leaving the resting membrane potential unchanged (Fig. 3*D*; spike number, two-way RM ANOVA, input current effect *p* = 0.0062, ACSF/OXT effect *p* = 0.0531, input current × ACSF/OXT interaction *p* = 0.0687; rheobase, two-tailed paired *t* test, *p* = 0.0182; resting membrane potential, two-tailed paired *t* test, *p* > 0.9999). Oxytocin application did not result in significant changes in excitability or membrane potential of DRN^DA^ neurons (Fig. 3*E*; spike number, two-way RM ANOVA, input current effect *p* = 0.0008, ACSF/OXT effect *p* = 0.2522, input current × ACSF/OXT interaction *p* = 0.7251; rheobase, two-tailed paired *t* test, *p* = 0.4018; resting membrane potential, two-tailed paired *t* test, *p* = 0.4837). The increased activity of DRN GABAergic neurons raises the possibility that local inhibitory circuitry contributes to the suppression of DRN^SERT^ neurons (Gervasoni et al., 2000; Liu et al., 2000), whereas the increased c-Fos expression in DRN^DA^ neurons despite the absence of changes in intrinsic excitability suggests that synaptic mechanisms or other local network effects may contribute to dopaminergic activation. These interpretations remain indirect, and the present experiments do not establish the specific circuit relationships among these populations.

**Figure 3.**
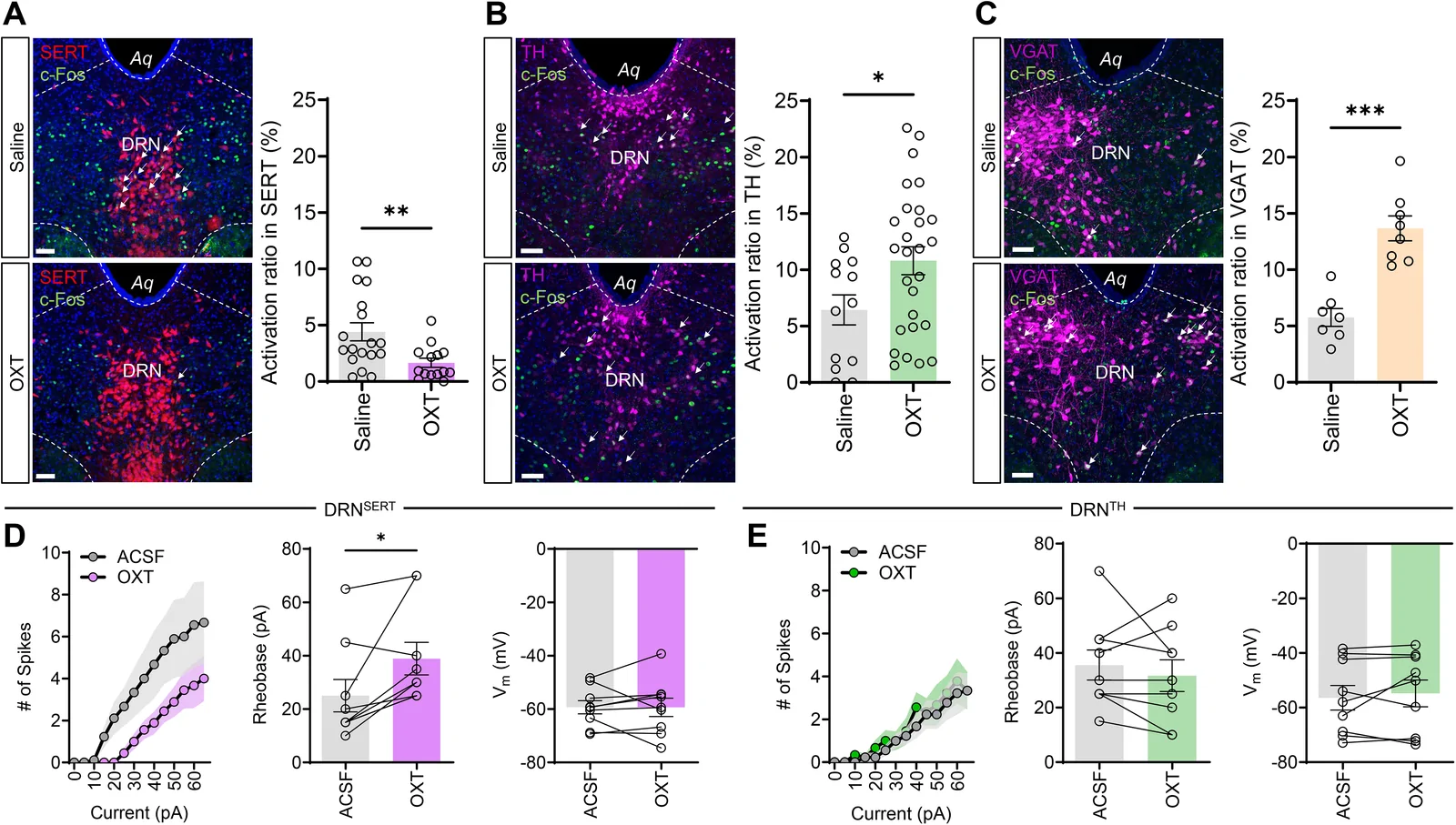
Bidirectional modulation of oxytocin in serotonergic, dopaminergic, and GABAergic neurons in DRN. ***A***, Representative images of c-Fos expression in serotonergic population in the DRN. The activation ratio is calculated as the percentage of SERT neurons positive for c-Fos (saline, *n* = 18 slices from 6 mice; OXT, *n* = 14 slices from 5 mice). Scale bars, 50 µm. ***B***, Representative images of c-Fos expression in dopaminergic population within the DRN. The activation ratio is calculated as the percentage of TH neurons positive for c-Fos (saline, *n* = 13 slices from 3 mice; OXT, *n* = 27 slices from 6 mice). Scale bars, 50 µm. ***C***, Representative images of c-Fos expression in GABAergic population within the DRN. The activation ratio is calculated as the percentage of VGAT neurons positive for c-Fos (saline, *n* = 7 slices from 2 mice; OXT, *n* = 8 slices from 2 mice). Scale bars, 50 µm. White arrows in ***A***–***C*** indicate c-Fos-positive cells colocalized with the corresponding neuronal marker. ***D***, The responses of DRN^SERT^ upon bath application of oxytocin. Number of spikes across current injections, rheobase, and resting membrane potential are shown (*n* = 9 neurons). ***E***, The responses of DRN^DA^ upon bath application of oxytocin. Number of spikes across current injections, rheobase, and resting membrane potential are shown (*n* = 9 neurons).

## Discussion

Our study observed the bidirectional effects of oxytocin on the neuronal activity of serotonergic and dopaminergic neurons in the DRN. Through anatomical visualization, we found varicosities as the contact sites between PVN^OXT^ and both DRN^SERT^ and DRN^DA^ populations. Furthermore, administration of oxytocin receptor antagonists into the DRN resulted in a reduction in social interaction levels, suggesting the role of oxytocin signaling in DRN mediating social behavior.

Previous studies have elucidated oxytocinergic neurons in the PVN project to the DRN, where serotonergic neurons and dopaminergic neurons are located (Weissbourd et al., 2014; Huang et al., 2019). Electron microscopic analysis has indicated the expression of oxytocin receptors at the synapses and on the axon (Mitre et al., 2016), suggesting that oxytocin can influence neural circuits through diverse cellular arrangements. Our study builds upon these findings by employing the dual-eGRASP tool, allowing us to visualize the specific contact sites of PVN oxytocinergic neurons with serotonergic and dopaminergic neurons, primarily at varicosity-like structures. Notably, the GRASP puncta at these connections were relatively sparse and differed from the distributions observed in other circuit combinations, including glutamatergic to glutamatergic (Choi et al., 2018, 2021; Lee et al., 2023), glutamatergic to GABAergic (Kim et al., 2024), and dopaminergic to medium spiny neurons (Choi et al., 2022), where puncta are more commonly associated with spines and dendritic shafts. Because oxytocin release from axonal varicosities has been reported in other neural circuits, this organization may reflect a structural substrate for local oxytocin signaling in the DRN (Oti et al., 2021; Qian et al., 2023). Given the sparse distribution of the GRASP signal and the neuromodulatory nature of oxytocin signaling, puncta density may not provide a reliable quantitative measure of connection strength. We therefore interpret the dual-eGRASP data primarily as anatomical evidence supporting putative contacts between PVN oxytocinergic neurons and serotonergic or dopaminergic neurons in the DRN. However, dual-eGRASP labeling alone does not determine whether these contacts represent synaptic release sites or contribute to nonsynaptic volume transmission. Further ultrastructural and functional analyses will therefore be required to define how PVN-derived oxytocin acts on serotonergic and dopaminergic populations in the DRN.

Our finding that oxytocin suppresses DRN^SERT^ neuronal activation and excitability differs from previous studies reporting facilitatory effects of oxytocin on serotonergic signaling. In the nucleus accumbens, oxytocin was shown to promote serotonin-dependent synaptic plasticity associated with social reward (Dölen et al., 2013). In addition, a previous study reported excitatory effects of oxytocin on DRN serotonergic neurons (Oubraim et al., 2023). Both our study and Oubraim et al. (2023) examined acute DRN slices with bath-applied oxytocin at 1 µM but differed in species, age, cell-identification strategy, and electrophysiological readouts. Oubraim et al. used young male rats and identified putative serotonergic neurons based on electrophysiological properties with post hoc Tph2 validation, whereas we recorded from genetically identified SERT neurons in adult SERT::Cre mice. These differences, together with heterogeneity among DRN serotonergic populations, may contribute to the contrasting effects observed between the two studies. Notably, although the oxytocin receptor is canonically Gq-coupled, OXTR can engage multiple G-protein signaling pathways depending on cellular context (Jurek and Neumann, 2018). Because our slice recordings were performed with bath-applied oxytocin in the absence of synaptic blockers, we cannot distinguish whether the observed suppression of DRN^SERT^ excitability reflects a direct postsynaptic OXTR-mediated effect or local circuit-mediated inhibition. Notably, systemic oxytocin administration increased c-Fos activation in DRN GABAergic neurons, raising the possibility that recruitment of local inhibitory circuitry contributes to the suppression of DRN^SERT^ neurons. This interpretation is consistent with electrophysiological, optogenetic, and viral-mediated tracing studies showing that local GABAergic circuitry provides inhibitory input to DRN serotonergic neurons (Gervasoni et al., 2000; Liu et al., 2000; Challis et al., 2013; Weissbourd et al., 2014; Conde et al., 2025). However, the present data do not establish a causal relationship between GABAergic activation and serotonergic inhibition. Other potential mechanisms may include alternative G-protein coupling of OXTR in specific cellular contexts or downstream modulation of membrane conductances that reduce neuronal excitability (Gong et al., 2015; Pati et al., 2020). Future experiments using synaptic blockers, measurements of excitatory and inhibitory synaptic currents, and pharmacological dissection of OXTR signaling will be required to distinguish among these possibilities and determine how oxytocin suppresses DRN^SERT^ neuronal activity.

Oxytocin neurons in the PVN and serotonergic and dopaminergic neurons in the DRN are all well-established mediators of prosocial behavior. Thus, the bidirectional neuromodulation by oxytocin observed here—specifically, the in vivo inhibition of serotonergic neurons alongside the activation of dopaminergic neurons within the DRN—is somewhat counterintuitive. Why would neuronal populations that each promotes prosocial outcomes possess a mechanism to inhibit one another? We hypothesize that this bidirectional regulatory network serves as a homeostatic buffer for social behavior. Specifically, rather than mutually reinforcing one another to drive extreme prosocial states during routine contexts like social approach, these neuromodulatory systems may leverage bidirectional modulation to maintain an optimal, balanced level of social engagement. Whether a similar bidirectional neuromodulation operates under different internal or physiological states during social interactions remains an intriguing open question. For instance, the expression and regulation of maternal behavior—a much more robust form of prosocial behavior than simple social approach—is known to involve not only PVN oxytocinergic signaling within the DRN as well as serotonergic and dopaminergic systems (Leff-Gelman et al., 2025). The allostatic shift in social behavior triggered by social isolation can also be considered as such example (Matthews and Tye, 2019; Choi et al., 2022). Together, the intricate interplay among PVN oxytocin neurons and DRN dopaminergic and serotonergic systems uncovered in this study would provide a conceptual framework for comprehensively understanding a wide range of social interactions.

## Synthesis

Reviewing Editor: Ayako Watabe, The Jikei University School of Medicine Decisions are customarily a result of the Reviewing Editor and the peer reviewers coming together and discussing their recommendations until a consensus is reached. When revisions are invited, a fact-based synthesis statement explaining their decision and outlining what is needed to prepare a revision will be listed below. The following reviewer(s) agreed to reveal their identity: Hiroshi Nomura.

This manuscript elucidates the anatomical and functional mapping of the PVN-to-DRN oxytocin circuit, showing how it bidirectionally modulates serotonergic and dopaminergic neurons during social behavior. Utilizing dual-eGRASP and ex vivo electrophysiology, the study successfully demonstrates a cell-type-specific mechanism.

　Overall, this is a well-conceived study with strong potential. Both reviewers and I agree that clarifying this distinct pathway represents a significant conceptual advance in understanding neuromodulation in social behavior. To ensure the conclusions are fully substantiated, the reviewers requested addressing the following points to further enhance the rigor and impact of this manuscript.

Specific Comments

1.

To fully solidify the behavioral findings and rule out any potential off-target effects of CNO alone, it is highly recommended to include an mCherry-only control group treated with CNO for Figure 1.

2.

The text mentions locomotor activity data in relation to Figure 1D, but the corresponding panel appears to be missing from the current layout. Please include this data to confirm that social deficits are distinct from general motor impairment.

3.

For Figure 2E-I, please specify the sample sizes (number of mice and brain sections) and perform control experiments omitting individual AAV constructs to confirm signal specificity.

4.

While a significant difference is shown in Figure 2B, providing the raw data prior to subtraction would offer a more comprehensive understanding of the results.

5.

The sample sizes for the behavioral assays (e.g., n=3-4 in Figure 2B and C) are somewhat small, which may limit statistical power. To maximize the rigor of these findings, incorporating a quantitative analysis of the puncta in Figure 2E-I would strengthen the manuscript.

6.

The manuscript notes an increase in c-Fos expression in DA neurons following systemic oxytocin administration, but no changes in intrinsic excitability in slice recordings, attributing this to local network effects. In the absence of direct experimental evidence (such as mEPSC/mIPSC recordings), providing a more detailed and nuanced discussion on the potential underlying mechanisms of these network effects would enrich the paper. Additionally, the electrophysiological data demonstrates that oxytocin suppresses the excitability of SERT neurons ex vivo. Because the oxytocin receptor is canonically Gq-coupled, this inhibitory effect is intriguing. While experimental identification of the exact molecular pathway would be a valuable addition, the authors are encouraged to expand the discussion to address potential pathways (e.g., indirect inhibition via local GABAergic interneurons, alternative G-protein coupling, or downstream Ca2+-activated K+ channels).

7.

The findings in Figures 3A-C present an interesting contrast to those reported by Oubraim et al. (2023). Given that this concerns a central claim of the study, the manuscript would benefit from an expanded discussion that explicitly clarifies which experimental conditions were comparable between the two studies and which differed.
